# Two Desmin Gene Mutations Associated with Myofibrillar Myopathies in Polish Families

**DOI:** 10.1371/journal.pone.0115470

**Published:** 2014-12-26

**Authors:** Jakub Piotr Fichna, Justyna Karolczak, Anna Potulska-Chromik, Przemyslaw Miszta, Mariusz Berdynski, Agata Sikorska, Slawomir Filipek, Maria Jolanta Redowicz, Anna Kaminska, Cezary Zekanowski

**Affiliations:** 1 Department of Neurodegenerative Disorders, Mossakowski Medical Research Centre, Polish Academy of Sciences, Warszawa, Poland; 2 Department of Biochemistry, Nencki Institute of Experimental Biology, Polish Academy of Sciences, Warszawa, Poland; 3 Department of Neurology, Medical University of Warsaw, Warszawa, Poland; 4 Faculty of Chemistry and Biological and Chemical Research Centre, University of Warsaw, Warszawa, Poland; 5 Neuromuscular Unit, Mossakowski Medical Research Centre, Polish Academy of Sciences, Warszawa, Poland; University of Vienna, Max F. Perutz Laboratories, Austria

## Abstract

Desmin is a muscle-specific intermediate filament protein which forms a network connecting the sarcomere, T tubules, sarcolemma, nuclear membrane, mitochondria and other organelles. Mutations in the gene coding for desmin (*DES*) cause skeletal myopathies often combined with cardiomyopathy, or isolated cardiomyopathies. The molecular pathomechanisms of the disease remain ambiguous. Here, we describe and comprehensively characterize two *DES* mutations found in Polish patients with a clinical diagnosis of desminopathy. The study group comprised 16 individuals representing three families. Two mutations were identified: a novel missense mutation (Q348P) and a small deletion of nine nucleotides (A357_E359del), previously described by us in the Polish population. A common ancestry of all the families bearing the A357_E359del mutation was confirmed. Both mutations were predicted to be pathogenic using a bioinformatics approach, including molecular dynamics simulations which helped to rationalize abnormal behavior at molecular level. To test the impact of the mutations on *DES* expression and the intracellular distribution of desmin muscle biopsies were investigated. Elevated desmin levels as well as its atypical localization in muscle fibers were observed. Additional staining for M-cadherin, α-actinin, and myosin heavy chains confirmed severe disruption of myofibrill organization. The abnormalities were more prominent in the Q348P muscle, where both small atrophic fibers as well large fibers with centrally localized nuclei were observed. We propose that the mutations affect desmin structure and cause its aberrant folding and subsequent aggregation, triggering disruption of myofibrils organization.

## Introduction

Myofibrillar myopathies (MFMs, MFM) are a clinically and genetically heterogeneous group of disorders characterized by ectopic protein aggregates and myofibrillar disorganization. The disintegration of the myofibrils commences in the proximity of the Z-disk. This is followed by abnormal protein expression and accumulation of degraded filamentous material in various patterns in myofibril-free fiber regions around nuclei and under the sarcolemma. Degradation of dislocated membranous organelles in autophagic vacuoles can also be observed [1].

The clinical features of MFM are diverse. Patients usually present with progressive muscle weakness that begins in distal muscles and spreads proximally, but in some cases an early limb-girdle involvement can occur. Cardiomyopathy is a frequently associated symptom [2]. The age at onset ranges from infancy to the eighth decade of life, but for the majority of cases the symptoms appear in the fourth and fifth decades [3]. Because of the significant phenotypic and pathomorphological variability, the diagnosis of MFM is often difficult.

An increasing number of genes are reported to be involved in MFM pathogenesis, causing subtypes of the disease[1]. Until now eight MFM-causing genes encoding Z-disc associated proteins have been identified: desmin (*DES*), αB-crystallin (*CRYAB*), myotilin (*MYOT*), Z band alternatively spliced PDZ-containing protein (*ZASP*), filamin C (*FLNC*), Bcl2-associated athanogene-3 (*BAG3*), four-and-a-half LIM protein-1 (*FHL1*) and titin (*TTN*) [4]–[8]. MFM are usually transmitted in an autosomal dominant manner, however, autosomal recessive or X-linked MFM forms have also been described [9].

Desmin (DES) is a helical muscle-specific main structural intermediate filament (IF) protein forming a network connecting Z-disk, T tubules, sarcolemma, nucleus, mitochondria and other organelles. It is expressed in skeletal, cardiac and smooth muscles, and has been proposed to be responsible for mechanochemical signaling and intracellular transport [10]. As a scaffold-provider it interacts with numerous proteins, such as α-actinin and actin (Z-disk proteins), nebulin (thin filament protein), MLH1 (mismatch repair protein), myospryn (protein involved in vesicular trafficking), desmoplakin (protein associated with desmosomes), dysferlin (protein implicated in calcium-dependent sarcolemma resealing), and myotubularin, a postulated regulator of the cytoskeleton architecture.

Mutations in *DES* were the first to be described in MFM cases [11], [12]. The gene maps to 2q35, comprises 9 exons spanning 8.4 kb, and encodes a 470-amino acid protein [13], [14]. Desmin is composed of an extended α-helical rod of 303 amino acid residues flanked by N-and C-terminal globular structures. The rod domain features a heptad repeat pattern which allows two helices to twist around each other yielding a homopolymeric coiled-coil dimer, the elementary unit of the filament. Interruptions in this pattern create short, non-helical linkers that connect four consecutive α-helical segments, 1A, 1B, 2A and 2B [15].

The coiled-coil segment 2B of all intermediate filament proteins contains a conserved discontinuity of the heptad repeat called a “stutter” (356-359), which is equivalent to an insert of four residues and is critical for IF growth.

Sixty-seven *DES* mutations have been described so far, but the correlation between particular mutations and the clinical phenotype is poorly understood [16], [17].

We have conducted a genetic study of three Polish families in which 16 individuals are affected with skeletal myopathy. The study includes pedigree analysis, mutation detection, immunostaining of biopsies and molecular modelling of desmin protein.

## Materials and Methods

### Patients

Members of three families with a clinical diagnosis of MFM were analyzed (for details see: S1 Table). The families were not known to be related.

### Family DP

**Figure 1:**
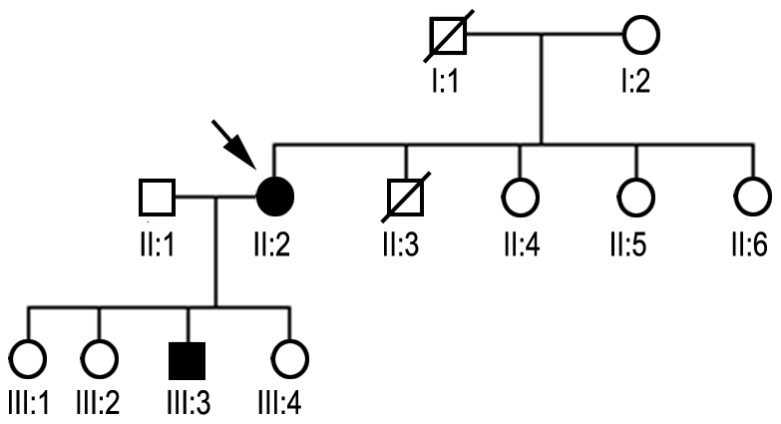
Pedigree diagram of family DP. The proband is marked with an arrow. Black symbols indicate clinically affected family members, open symbols indicate unaffected, and crossed symbols indicate deceased persons. Family DP consisted of twelve members, of which only one participated in the study. Genetic analyses were performed for II:2 (the proband) and III:3 (proband's son).

The patient (III:3) is a 24-year-old Caucasian male. At age 21, he developed progressive atrophy of the lower limb muscles followed by lower limb weakness coexisting with fasciculation and painful cramps especially after exercise. His family history was positive, similar symptoms occurred in his mother (Fig. 1). Neurological examination revealed high arched palate, weakness and significant wasting of the lower limbs muscles. Deep tendon reflexes were symmetrical in the upper limbs and completely diminished in lower limbs. Patient was unable to walk on his heels or on his toes, and needed support during knee bending. Physical examination revealed overweight (probably due to diminished activity) and gynecomasty.

No abnormalities were found in laboratory examinations including hematology and hormone levels (TSH, fT3, fT4, prolactin, testosterone and cortisol). Results of a biochemical test panel including lipidogram were within norm except for a significant elevation of serum creatine-kinase (CK) activity (580 IU, normal values: 0–34 IU). No abnormalities were found in brain MRI (including pituitary gland), electroencephalography (EEG), and electrocardiography (ECG) was also normal.

The electromyogram (EMG) of the right biceps and right vastus lateralis muscles showed myopathic features (decreased duration and size index of motor unit action potentials, MUAP). Nerve conduction was normal.

An open muscle biopsy of the left quadriceps muscle was performed. A morphological analysis of the biopsies showed moderate myopathic changes. On light microscopy with HE staining the fascicular architecture of the muscle showed a marked variability in fiber size and shape, with some atrophy of the fibers partially preserved as “nuclear clumps”. Trichrome staining revealed in some of fibers with small pink colored or dark inclusions. Enzymatic reactions showed preserved metabolic differentiation of the fibers but marked atrophy of type 2 fibers was observed as well as numerous fibers with a lobulated structure.

The patient's mother (proband) is 47 years old. At the age of 40 she developed gait disorders due to lower limb weakness with drooping left foot. She also suffered from lower back pain. She was diagnosed as L/S discopathy with laminectomy three years ago, and has a medical history of hyperthyroidism. Neurological examination revealed high arched palate, nasal speech, weakness and wasting of the muscles of the lower limbs (L>R). Deep tendon reflexes were symmetrical in the upper limb. Right knee reflex was decreased and left was absent. Both Achilles reflexes were strongly diminished. Patient was unable to walk on her heels or on her toes, and needed support during knee bending.

Laboratory examinations revealed normal hematological parameters. Results of a biochemical test panel including lipidogram were within norm except for an elevation of serum CK activity to 78 IU.

No cataracts were found by ophtalmoscopy. Fundus of the eye was normal. ECG and chest X-ray were normal. Echocardiogram (EchoCG) was normal with EF over 70%.

An EMG of the right tibialis anterior and right vastus lateralis muscles showed myopathic features i.e. decreased duration and size index of MUAP (motor unit action potential). Nerve conduction was normal except for a decrease of M-response in both peroneal nerves presumably due to atrophy of the muscles.

### Family ZP

**Figure 2:**
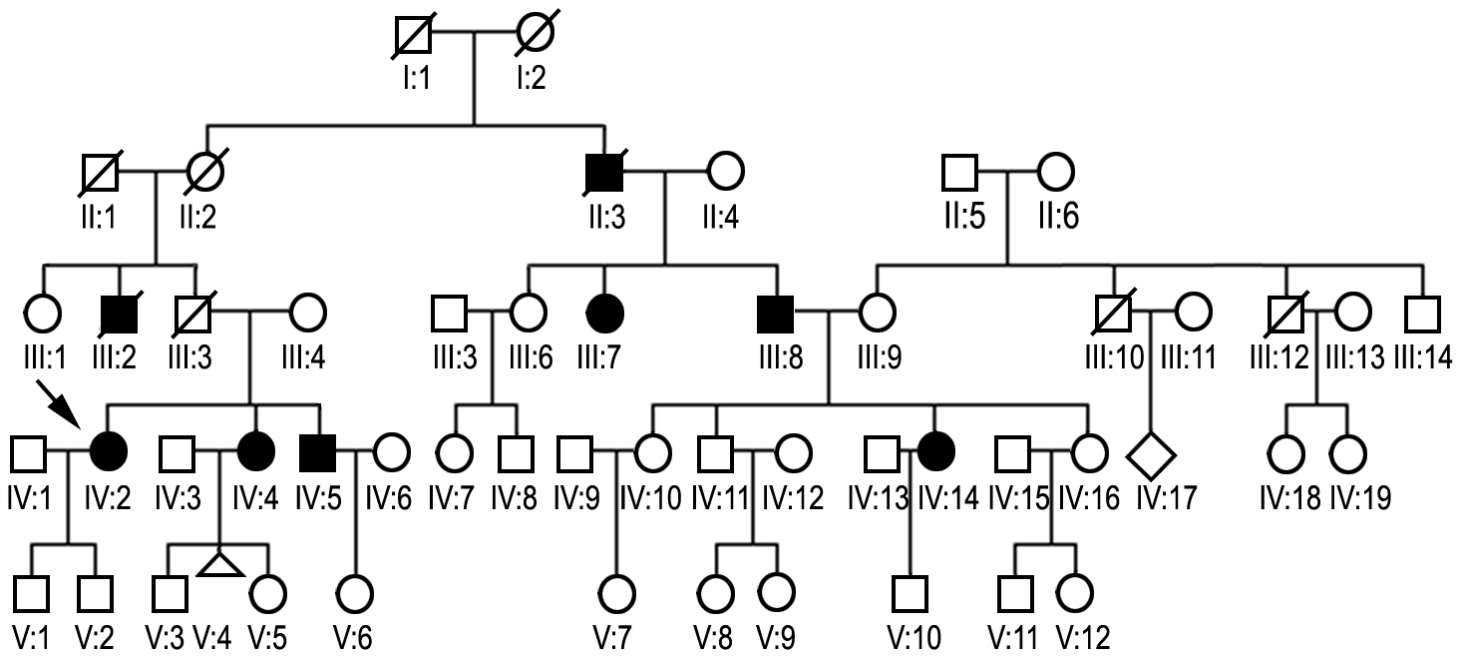
Pedigree diagram of family ZP. Open diamond (IV:17). indicates sex unknown. Open triangle (V:4) indicates miscarriage. The other symbols indications as above. Family ZP consisted of 52 members, five of which participated in the study. Genetic analyses were performed for individuals III:7, IV:2, IV:4, IV:5, and IV:14.

The disease onset in the affected members of the family (proband, her sister and their father's brother, who died of heart failure at the age of 48) was around 40 years of age, with progressive lower limb weakness, difficulty in climbing stairs and occasional falls (Fig. 2).

Neurological examination of the proband (IV:2) revealed high arched palate, slight atrophy of the left thenar muscle, weakness and wasting of the lower limbs muscles L>R, most pronounced in the proximal part of the legs. Deep tendon reflexes were symmetrical in the upper and lower limbs, and only slight diminishing of both knee reflexes was observed. The patient was unable to walk on heels and needed some support during knee bending.

Laboratory examinations revealed normal hematological parameters. Results of a biochemical test panel including lipidogram and lactic acid were within norm except for an elevation of serum CK activity to 81 IU.

An EMG of the right biceps and right vastus lateralis muscles showed myopathic features with decreased duration and size index of MUAP. Nerve conduction was normal except for a decreased M-response amplitude to left median nerve stimulation (presumably due to the observed atrophy of the left thenar muscle).

An open muscle biopsy was performed in the proband. Its morphological analysis showed moderate myopathic changes of the left quadriceps muscle. On light microscopy with HE staining the fascicular architecture of the muscle showed a marked variability in fiber size and shape, with some atrophy of the fibers partially preserved as “nuclear clumps”. Several fibers had multiple centrally located nuclei. Trichrome staining revealed pink colored inclusions in a few fibers. In the enzymatic reactions metabolic differentiation of the fibers was preserved with only slight atrophy of type 2 fibers.

### Family KP

**Figure 3:**
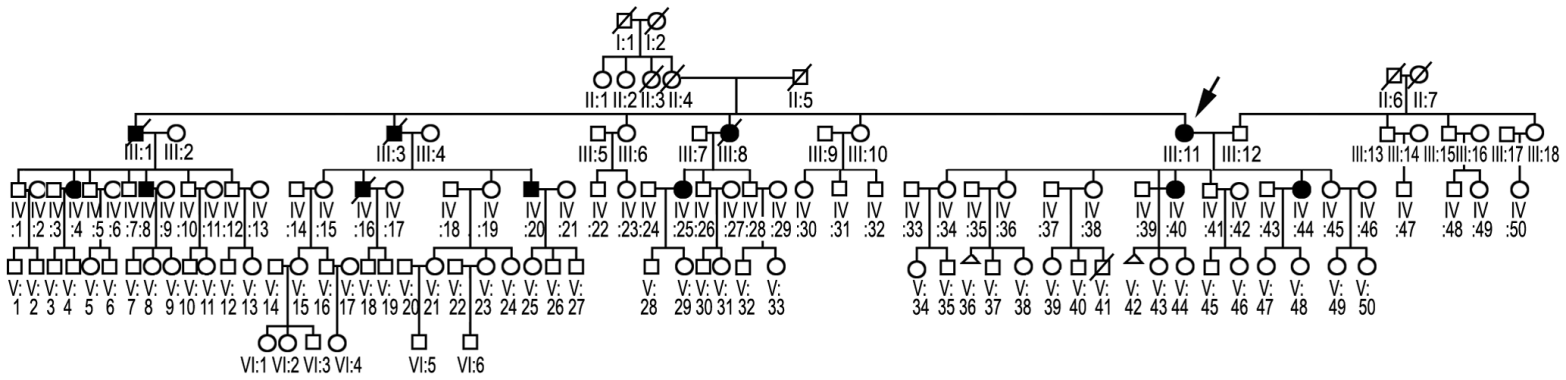
Pedigree diagram of family KP. The symbols indications as above. Family KP consisted of 183 members, and eleven participated in the study. Genetic analyses were performed for III:1, III:11, IV:20, IV:25, IV:40, IV:44, V:7, V:8, V:9, V:43, V:44.

**Figure 4:**
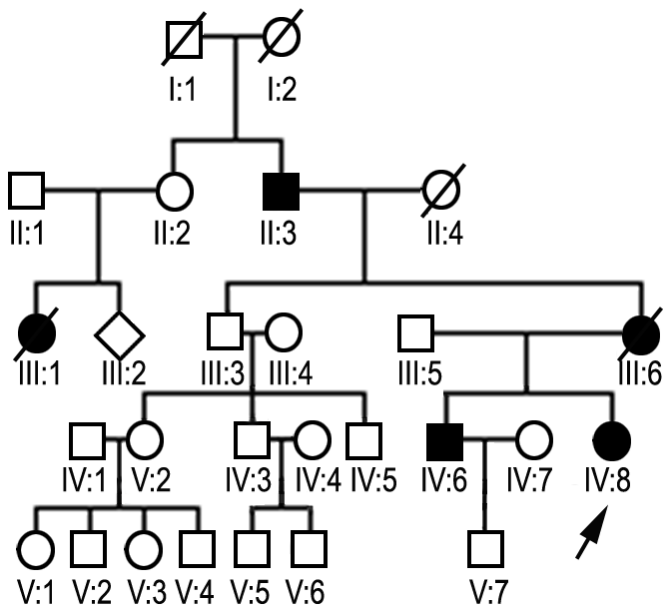
Pedigree diagram of family RP. The symbols indications as above. Family RP consisted of 27 members, three of which (IV:6, IV:8, V:7) participated in the previous study [18].

The symptoms in the affected members of the family (Fig. 3) were similar to those in a previously reported family RP (Fig. 4) [18]. The disease onset with gait disturbance and bilateral weakness in the lower limbs was between 39 and 45 years of age. The mother and daughter first developed weakness in the proximal and later in the distal leg muscles, whereas the niece had this sequence reversed. Weakness and wasting slowly spread to other muscle groups of the lower extremities and eventually involved the upper limbs. The mother became wheelchair-dependent 18 years after the disease onset. On examination, each patient had moderate weakness and wasting in the lower and upper extremities, except for the daughter who showed only mild symmetrical weakness in the proximal leg muscles. Tendon reflexes were preserved, no sensory deficits were detected. ECG and EchoCG studies were unremarkable. An EMG showed a myopathic pattern in each patient but was normal in the daughter. Motor and sensory conduction velocities were normal. Serum CK levels were 2–7 times normal ( 69–225 IU). Sections of skeletal muscle biopsy stained with the Gomori's trichrome method showed reddish cytoplasmic inclusions which were reactive for desmin in immunostaining. Electron microscopic evaluation showed abnormal granulofilamentous aggregates among the myofibrils.

The new patient from the KP family is a 49 year old female (IV:44). At age 40, she developed progressive proximal weakness of the lower limbs followed by muscle atrophy. Since the age of 48 she occasionally experienced transient dyspnea after physical effort.

Neurological examination revealed weakness and significant wasting of the lower limb muscles (proximal and distal). Deep tendon reflexes were symmetrical in the upper limbs and absent in the lower limbs. She was unable to walk on heels or toes. Support was necessary during knee bending. She presented Gowers maneuver during standing up from the floor.

Results of a biochemical test panel including lipidogram were normal except for a slight elevation of serum CK activity (88 IU). Continuous, day-long ECG, and EchoCG were within norm and chest X-ray was also normal. EMG of the biceps, first dorsal interosseous, vastus lateralis and tibialis anterior muscles on the right side showed myopathic features with decreased duration and size index of MUAP. Nerve conduction was normal.

Additionally, MRI of the hamstring muscles showed characteristic patterns of predominant involvement of the semitendinosus and iliopsoas muscles with relative sparing of the semimembranosus (S1a Fig. and S1b Fig.).

### Mutation screening

Patients (n = 11) and their unaffected family members (n = 5) were screened for the presence of *DES* mutations. Genomic DNA was isolated from peripheral blood leukocytes using standard methods. Intronic primers were used to amplify exons 1–9 of *DES*. Amplicons were purified with Exonuclease I/FastAP (Fermentas) and sequenced on an ABI PRISM 3130 Genetic Analyzer (Applied Biosystems) using the BigDye Terminator v1.1 Cycle Sequencing Kit (Applied Biosystems).

### Haplotype analysis of the deletion region of the gene

Genotyping of patients and unaffected members of the families with the A357_E359del mutation was performed using highly variable microsatellite (STR) markers flanking the mutation locus: D2S2382, D2S2248, D2S1338, D2S163, D2S126, D2S133, and D2S2354. PCR amplification of the STRs was performed using fluorescently labelled primers. The PCR-amplified fragments were analyzed using an ABI PRISM 3130 Genetic Analyzer, and sized using Gene Mapper software (Applied Biosystems). Additionally, intragenic single nucleotide polymorphisms (SNPs) at nucleotide positions 828 (rs1058261), 1014 (rs12920), and 1104 (rs1058284) of the desmin gene were genotyped (nucleotide and amino acid sequence data are available in the CCDS database under accession CCDS33383.1). The haplotype was determined based on pedigree analysis and sequencing results.

### Muscle biopsy

Open muscle biopsies of the quadriceps were performed and processed for further analyses. Muscle biopsies taken for diagnostic purposes from unrelated patients without morphological changes were used as controls.

### Antibodies and fluorescent markers

The following monoclonal mouse antibodies were used in the study: anti-human desmin (D 33, Dako), anti-mouse M-cadherin (611100; BD Biosciencies), anti-GAPDH (glyceraldehyde 3-phosphodehydrogenase; clone 6C5; Merck Millipore), anti-human slow myosin heavy chain (MHCs; NOQ7.5.4D) and anti-rabbit sarcomeric α-actinin-1; EA-53 (both from Abcam. For immunocytochemistry studies, goat anti-mouse IgG conjugated with AlexaFluor-546 (A-21236; Life Technologies). DAPI applied in the Vectashield mounting medium (Vector Labs) was used to stain nuclei. For immunoblot, anti-mouse IgG (#AP308P) conjugated with horse radish peroxidase (HRP), from Millipore was used.

### Electron microscopy analysis of the muscle biopsy

Part of the muscle specimens for electron microscopy were fixed in glutaraldehyde, postfixed in osmium tetroxide before embedding in Spurr embedding medium (Electron Microscopy Sciences, USA). Ultrathin sections were stained with uranyl acetate and counterstained with lead citrate. The samples were viewed with JEM 1200 EX2 electron microscope.

### Immunolocalization studies

Distribution of desmin, α-actinin (marker of Z-disc), M-cadherin (marker of adherens junction) and MHCs (constituent of thick filaments) in muscle biopsies obtained from patients with the A357_E359del (IV:2 from ZP family) and Q348P (III:3 from DP family) mutations, as well as a person without symptoms of muscle impairment were examined by indirect immunohistochemistry. Muscle cross-sections were fixed in 4% paraformaldehyde for 10 min. The fixed specimens were thoroughly washed in phosphate-buffered saline (PBS) and treated for 30 min with a solution of 5% normal goat serum and 0.2% Triton X-100 in PBS. Subsequently, muscle sections were incubated overnight at 4°C with the anti-desmin antibody, anti-α-actinin antibody, anti-M-cadherin or anti-MHCs (all at a dilution of 1∶100) followed by incubation with Alexa-546 conjugated anti-mouse secondary antibodies at a dilution of 1∶500 for 60 min at RT. The specimens were observed under a Leica TCS SP8 spectral confocal microscope equipped with an HCX PL APO 40x/1.25-0.75 Oil Cs objective. Optical sections (1024 pixels ×1024 pixels ×12 bits/pixel) were usually collected at 0.8 µm z-spacing. For negative controls, the primary antibody was omitted.

### Immunoblotting

Control (n = 4) and patients's muscles were homogenized in 10 volumes over the muscle mass of ice-cold PBS supplemented with 1 mM phenylmethylsulfonyl fluoride (PMSF) and Complete protease inhibitor cocktail (Roche Diagnostics GmbH). To evaluate the amount of desmin in muscle soluble fraction, the homogenates were centrifuged at 1800xg and supernatants were subjected for further analysis. Equal volumes of supernatants and homogenates of every sample were separated on 10% SDS-PAGE and transferred to a nitrocellulose membrane. The immunoreactive bands were detected by incubation with antibody against desmin and GAPDH followed by secondary antibody conjugated with horse radish peroxidase. The reaction was developed using ECL according to the manufacturer's instructions (Pierce). Developed blots were photographed and subjected to densitometric analysis using a G:Box system equipped with the GeneSnap and GeneTools software.

### Molecular modelling

The structure of desmin dimer (residues 333–411) was built using homology modelling tools (Swiss-Model server) [19] based on the structure of human vimentin coiled-coil 2B dimeric fragment (residues 328–406) deposited in the Protein Data Bank (id: 1GK4) [20]. The pairwise alignment of the two sequences contained no gaps due to their high similarity. The modifications, mutation Q348P and deletion A357-E359del, were introduced in both helices of the homology models of desmin dimer. The wild-type (WT) and modified structures were simulated in the box of TIP3P model [21] of water 4.1 nm ×4.2 nm ×13.5 nm holding an excess of about 1 nm of water layer between the protein and each box side. A cutoff of 1.2 nm was used with a switching function for calculations of non-bonded interactions. For each studied system 30-ns molecular dynamics (MD) simulations were conducted at 298^o^K and a pressure of 1 bar using the Nose-Hoover thermostat [22] and Langevin Piston pressure control [23] in the NAMD 2.9 program [24] employing CHARMM27 force field. A time step of 1 fs was used. Before the final MD simulations two-step equilibrations were performed: 1 ns MD with whole protein fixed and then 1 ns MD with the protein backbone fixed.

### Ethics Statement

Written consent was obtained from all patients and healthy individuals according to the Declaration of Helsinki (BMJ 1991; 302:1194). The genetic study was approved by the Ethics Committee of the Warsaw Medical University and MSW Hospital (Warszawa, Poland) in compliance with national legislation and the Code of Ethical Principles for Medical Research Involving Human Subjects of the World Medical Association. The same informed consent procedure was used for recruitment of the control group.

## Results

### Mutation screening

Two mutations in exon 6 of the *DES* gene were identified. In the sample from one patient from DP family a novel heterozygous Q348P (CAG > CCG, c.1043A >C) mutation was found. Patients from families ZP and KP showed the same heterozygous mutation A357_E359del (c.1076_1084del), already described in the Polish population (family RP) [18]. In all three families the mutations co-segregate with the disease. The exact location of the deletion is ambiguous due to the presence of a six-nucleotide repeat sequence (GCCAGT). The upstream repeat encodes residues A357–S358, whereas the downstream one codes for A360–S361. This alteration predicts an in-frame deletion of the E359–A360–S361 segment.

Bioinformatics analyses indicate that the Q348P mutation most probably causes desmin dysfunction leading to the MFM clinical phenotype. The Q348P mutation changes an uncharged, polar helix-maker glutamine residue into an a nonpolar helix-breaker proline residue in a region critical to desmin structure. Both mutations (Q348P and A357_E359del) alter the heptad periodicity within a critical, evolutionarily conserved coiled-coil domain 2B. Additionally, analysis using various programs, e.g.: ConSurf (http://consurf.tau.ac.il), SIFT (http://sift.bii.a-star.edu.sg), PROVEAN (http://provean.jcvi.org/index.php), Polyphen2 (http://genetics.bwh.harvard.edu/pph2/), and BLAST (http://blast.ncbi.nlm.nih.gov/Blast.cgi) demonstrated that the Q348 residue is highly conserved in evolution, and its replacement with a proline is deleterious. According to the criteria proposed by Cotton and Scriver [25], Antonarakis and Cooper [26], and the most recent ones by McArthur et al [27], it is highly probable that Q348P is a true disease-causing mutation.

### Origin of the deletion in the affected families

An extensive haplotype analysis performed in families KP and ZP and in one member from family DP (control) allowed pinpointing the STR haplotype connected with the A357_E359del mutation and shared by all the analyzed subjects (region D2S2382-D2S133). The results, combined with previous reports, strongly suggest a common ancestor of the apparently unrelated families ZP and KP, and the earlier studied RP family [18]. The small alterations from the disease-associated haplotype in one member of family KP (Table 1.) could be easily explained by a crossing-over event in the distal fragment of the analyzed *locus*. It should be noted that also D2S2354 is divergent in family ZP (262 or 276 bp), family KP (266 bp) and previously reported family RP (266 bp). An extended version of Table 1 is presented in the Supporting Information (S2 Table).

**Table 1:** Haplotype analysis of the families with the *DES* mutations.

	D2S2382	D2S2248	D2S1338	D2S163	rs1058261	rs12920	DES	rs1058284	D2S126	D2S133	D2S2354
Family ZP	308	182	238	222	C	G	Del	G	125	239	262/276
Family KP	308	182	238	222	C	G	Del	G	125	239	266
Family KP member IV:20	320/330	182/192	250/275								
Family RP (previously reported)	308	182	238	222	C	G	Del	G	125	239	266
Family DP member III:3	308/308	182/182	238/266	224/230	C/T	G/C	Q348P	G/A	141/147	239/239	262/264

Shared alleles in patients are shown. For each analyzed microsatellite marker the length (bp) of the PCR fragment shared among patients with the mutation is indicated.

### Muscle electronmicroscopy

**Figure 5:**
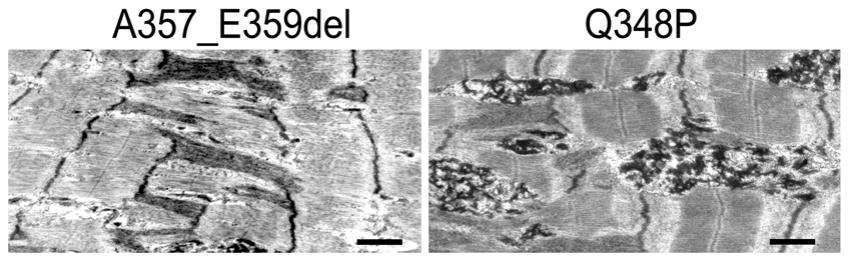
Electron microscopy analysis of the longitudinal sections of probands's muscle biopsies. Bars, 1 µm.

Electron microscopy showed dramatic changes in the fiber organization. In both muscle Z line widening/streaming was observed as well as desmin accumulation, especially prominent in the patient with Q348P mutation (Fig. 5).

### Immunostaining

**Figure 6:**
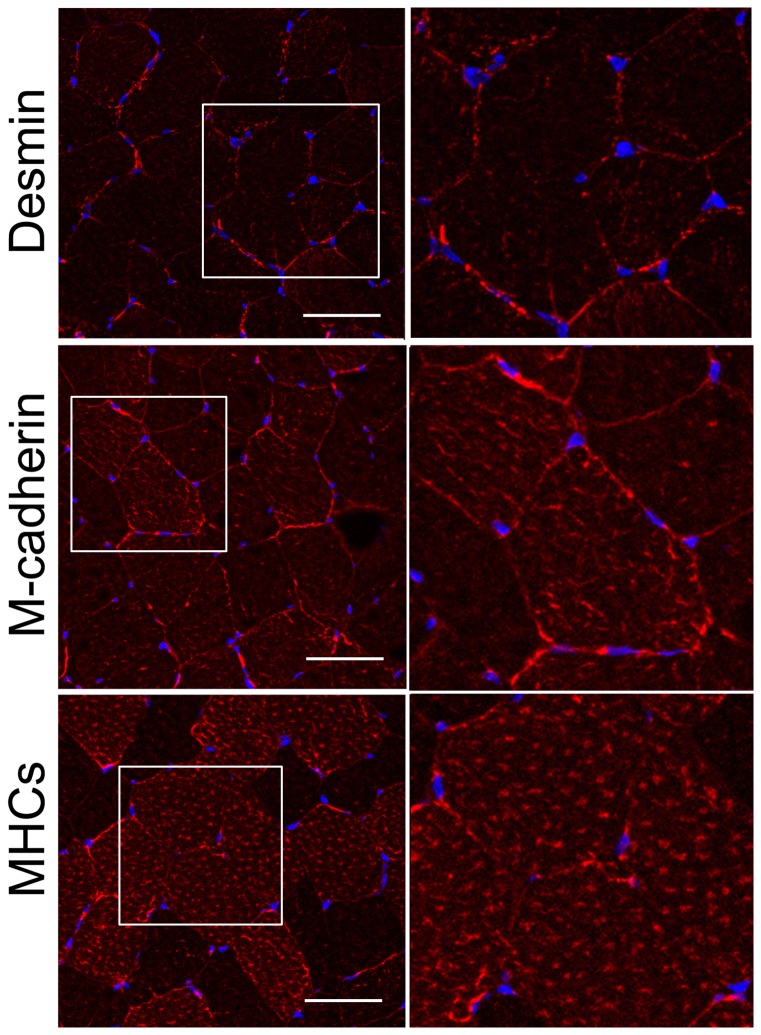
Distribution of desmin in control muscle. Transverse sections of muscle biopsy of a healthy subject were stained with anti-desmin, anti-M-cadherin and anti-slow myosin heavy chain (MHCs) antibodies, as indicated in the figure. Right panels, ∼2x magnification of the regions marked in left panels. These are 0.8-µm images of the center of transverses muscle section obtained with a Leica confocal microscope. Other details as described in Materials and Methods section. Bars, 50 µm.

**Figure 7:**
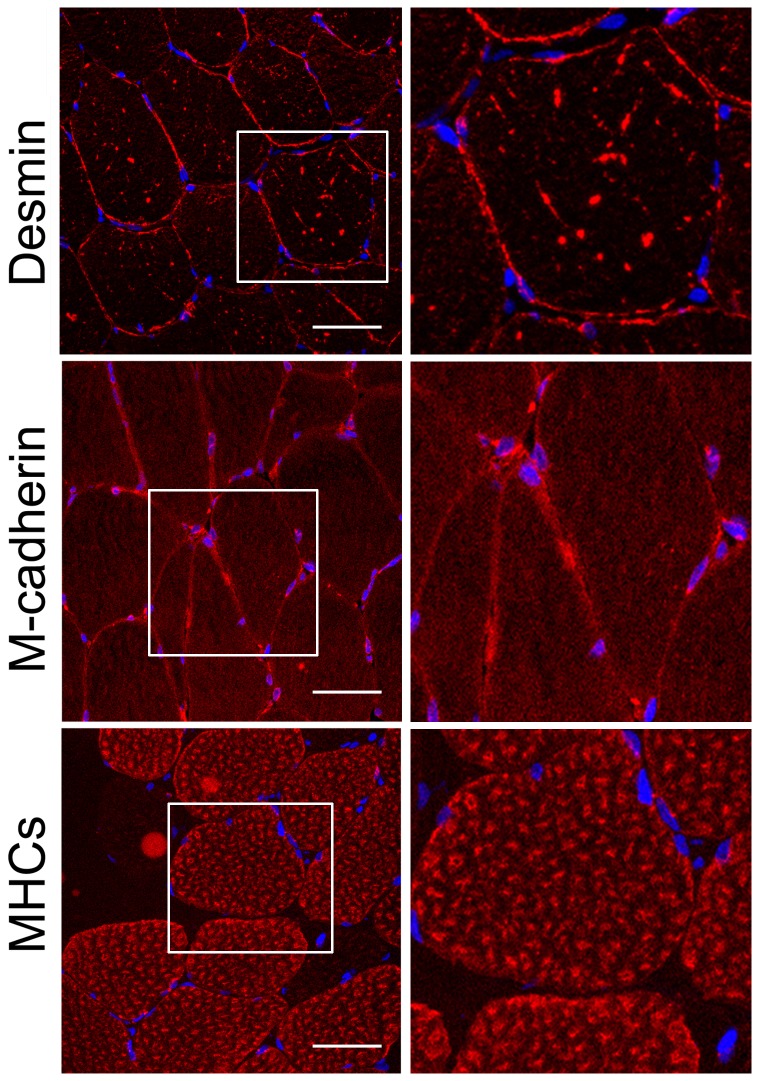
Distribution of desmin in muscle with A357_E359del mutation. Transverse sections of the muscle biopsy of the patient were stained with anti-desmin, anti-M-cadherin and anti-slow myosin heavy chain antibodies, as indicated in the figure. Right panels, ∼2x magnification of the regions marked in left panels. These are 0.8-µm images of the center of transverses muscle section obtained with a Leica confocal microscope. Other details as described in Materials and Methods section. Bars, 50 µm.

**Figure 8:**
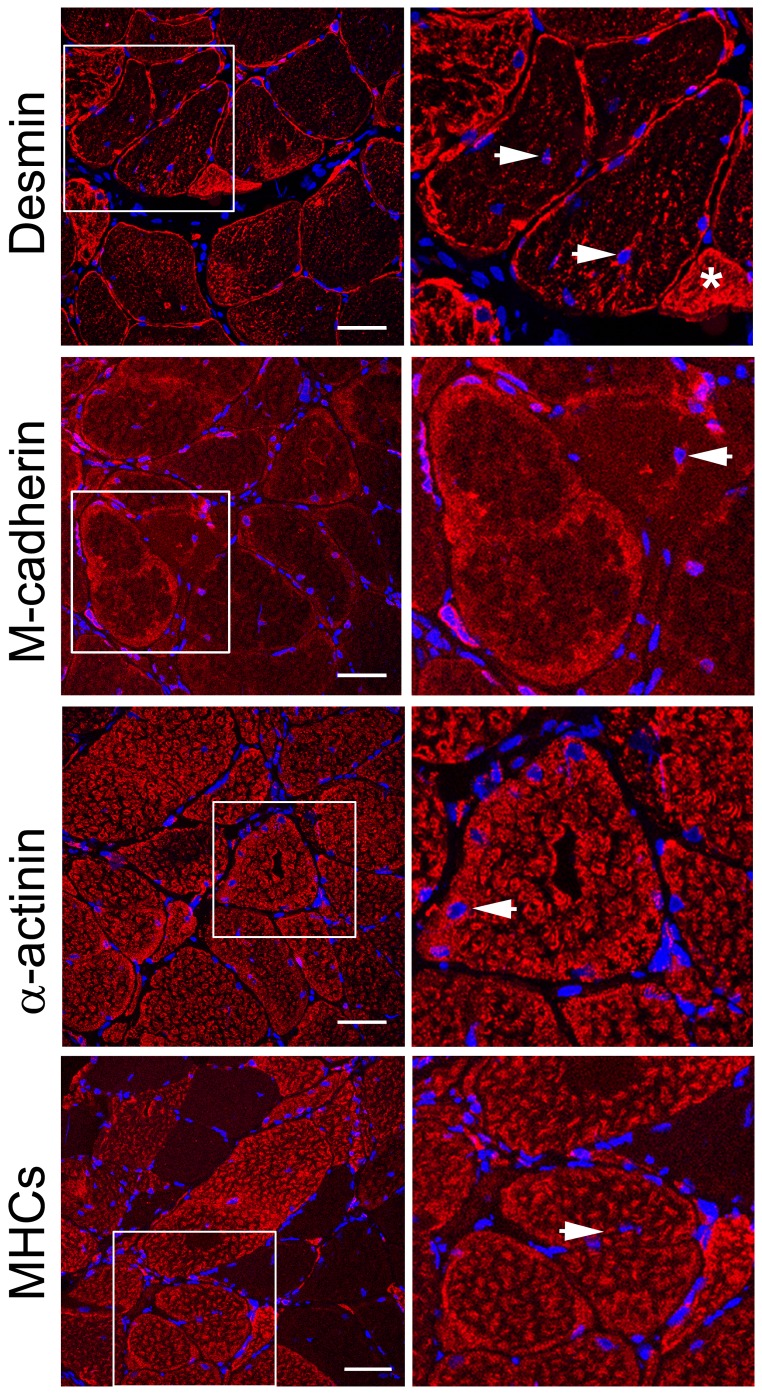
Distribution of desmin in muscle with Q348P mutation. Transverse sections of the muscle biopsy of the patient were stained with anti-desmin, anti-M-cadherin, anti-α-actinin and anti-slow myosin heavy chain antibodies, as indicated in the figure. Right panels, ∼2x magnification of the regions marked in left panels. Arrows, centrally located nuclei; asterisk, an atrophic fiber. These are 0.8-µm images of the center of transverses muscle section obtained with a Leica confocal microscope. Details as described in Materials and Methods. Bars, 50 µm.

To test whether the detected mutations in the desmin gene affect the intramuscular distribution of desmin and other muscle proteins, muscle biopsies from the control (C) and patients (IV:2 ZP family, A357_E359del and III:3 DP family, Q348P) were studied by immunostaining (Figs. 6–8).

As expected, in the transverse section of the control muscle desmin localized uniformly to the muscle periphery, and was also visible around Z-discs within the muscle fiber (Fig. 6). Staining for M-cadherin and MHCs also did not reveal any aberrations in the control muscle (Fig. 6).

Staining of the patients' muscle samples for desmin revealed an increase in the fluorescence intensity, especially for the samples of the patient with the Q348P mutation (see Fig. 7 and Fig. 8).

In the vast majority of muscle fibers with the A357_E359del mutation, desmin was present in numerous puncti-like or short elongated structures present within the fibers (Fig. 7). The ectopic desmin distribution was more prominent in the muscle with the Q348P mutation, where large desmin-containing filamentous or aggregate-like structures were abundant in large fibers (Fig. 8). Moreover, there were numerous centrally situated nuclei in these aberrant large fibers (Fig. 8, arrows). Also, more uniform desmin staining was observed in small atrophic myofibers (Fig. 8, asterisk).

Additional staining for α-actinin and MHCs also confirmed a disruption of myofibrillar organization, especially in the case of the Q348P mutation where the Z-discs and thick filaments seemed to be thicker and less uniformly organized (Figures 7 and 8). Also, more diffusive staining for M-cadherin was observed in the sample with A357_E359del mutation (Fig. 7) and not in the sample with Q348P mutation where it seemed to be accumulated in aggregate-like structures (Fig. 8).

### Quantification of desmin content

**Figure 9:**
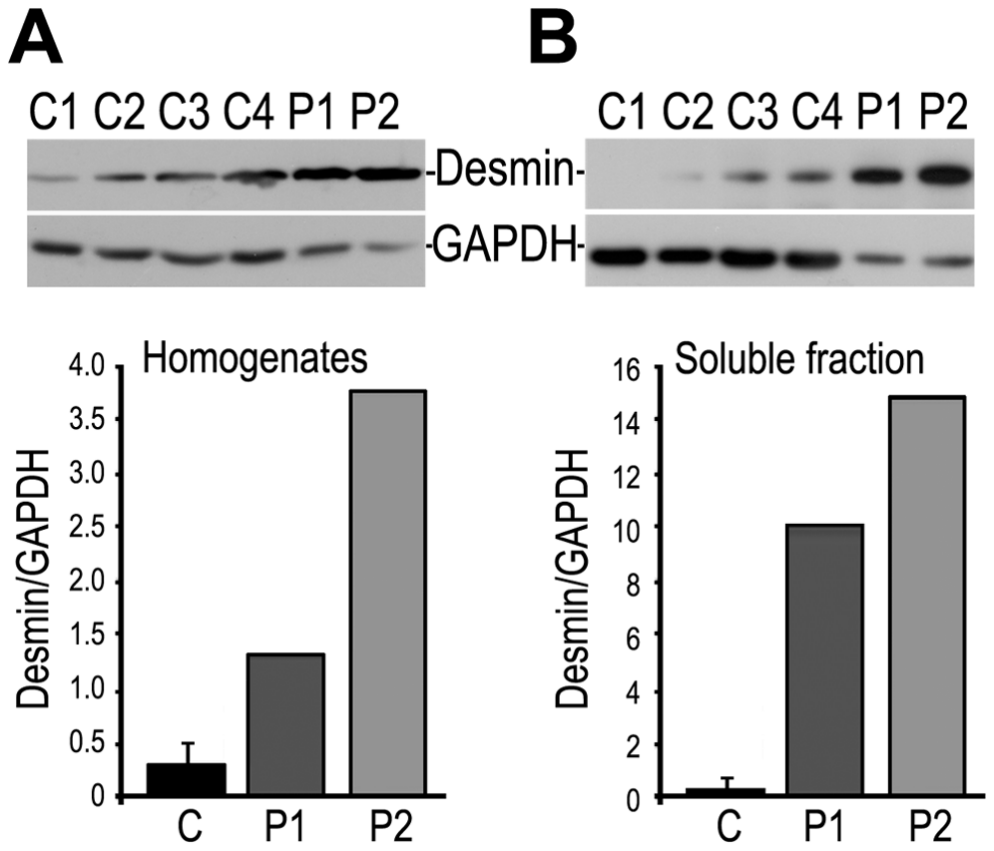
Quantification of desmin content in muscle by immunoblotting. Equal volumes of homogenates (A) and supernatants (B) from control (C1-C4), patient IV:2, ZP family (P1, A357_E359del mutation), and patient III:3, DP family (P2, Q348P mutation) muscle were subjected to SDS polyacrylamide gel electrophoresis, blotted on nitrocellulose membrane and probed with anti-desmin and anti-GAPDH antibodies, as described in Materials and Methods. Lower panels in A and B, densitometric analyses of desmin content in the examined muscles. For controls, the data are presented as mean ± SEM for n = 4.

To quantify the amount of desmin in the patients's muscle, we performed immunoblotting with anti-desmin antibody. The overall desmin content (with respect to the amount of GAPDH) in the patients' muscle homogenates (P1 and P2) was significantly higher than in control muscles (C1–C4; Fig. 9A). This increase was higher for P2 patient (with Q348P mutation) than for P1 patient (with the A357_E359del mutation).

Since immunostaining indicated that desmin in the patients' muscles could be not properly folded, we quantified the amount of desmin in the muscle soluble fraction. The immunoblot analysis revealed that in soluble fractions of patients's muscles a substantial amount of desmin was detected. Contrary to that, in some of control muscles the protein was barely detected there (Fig. 9B).

### Modelling

**Figure 10:**
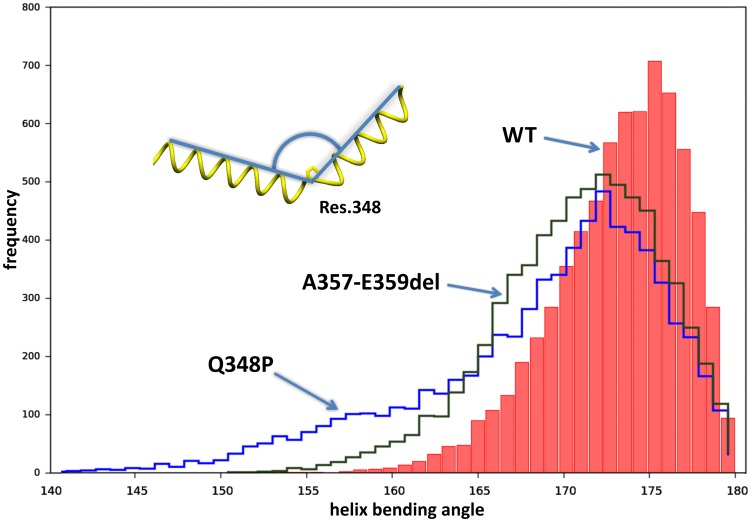
Histogram of helix bending angle at residue 348. WT helix is naturally bent, about 175°, in a dimer coiled-coil conformation. For the Q348P mutation the maximum of the helix bending angle is nearly the same, about 172°, but a long tail of this plot with a local maximum at 158° indicates a high flexibility of helix at this residue in the mutant structure. The structure of A357-E359del also demonstrates a higher flexibility than WT.

**Figure 11:**
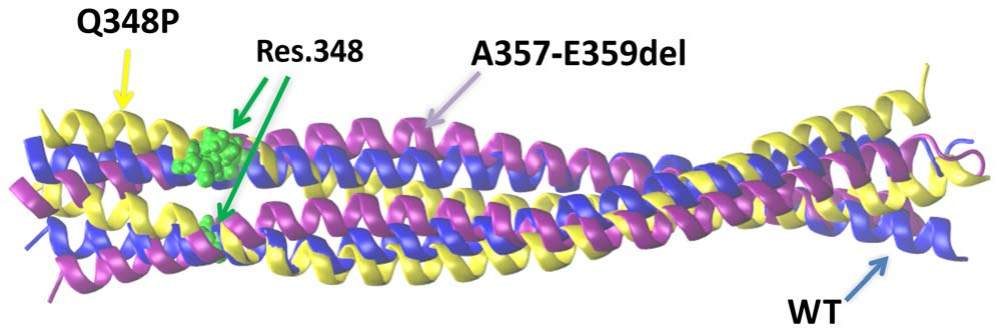
Alignment of WT (blue), point-mutated (yellow) and deletion (purple) structures of desmin dimer after a 30ns MD simulation. Residue 348 is colored green in both helices.

The helix in the wild type (WT) desmin is naturally bent (ca. 175°), due to the coiled-coil conformation. The WT desmin structure displays a range of helix bending angles (160°-180°) due to thermal motion. The Q348P mutation introduces high flexibility in the coiled-coil structure of the protein. The maximum of the helix bending angle in the mutated desmin is nearly the same as in WT, about 172°, but the range of possible angle is much larger, about 145°–180°. The local maximum on the plot line for Q348P (Fig. 10) at 158° indicates a secondary favored value of this bending angle (Fig. 11) which can be selected during formation of bent filament or even an aggregate. The structure of A357-E359del also demonstrates a higher flexibility than WT (Fig. 10) but not as high as for Q348P. The deletion introduces a mismatch between pairing residues of both helices which is reduced by partial unwinding the two coiled helices. Such unwinding leads to more straight (parallel) arrangement of both interacting helices (Fig. 11) in the region located close to the deletion.

A variable number of salt bridges was observed, depending on current state of MD simulation. The number was changing by about 50% in all simulations, as they were braking and creating again. However, the average number of salt bridges was the same in all analyzed cases.

## Discussion

Two mutations in the *DES* gene have been identified in three Polish families, with an autosomal dominant mode of inheritance. Haplotype analysis of patients from the families bearing the A357-E359del mutation revealed a common haplotype associated with the mutation, indicating a common ancestry. However, we have no access to sufficiently numerous group of family members, and population sample to perform an inferred age analysis placing the founder-mutation event in a particular period of time.

The clinical phenotypes observed in the reported families differed depending on the mutation. It seems that Q348P mutation causes more severe symptoms as compared to A357_E359del. Also, microscopic analyses performed with both electron and confocal microscopy of the muscle biopsies revealed a more dramatic effect of the point mutation on the myofiber organization as well as on desmin distribution and its assembly within the fibers. The fiber size was more diverse in patients' muscles, especially of the patient with the point mutation where both small atrophic fibers and large fibers with centrally situated nuclei were visible. Also, distribution of M-cadherin and sarcomeric proteins has been affected by desmin mutations thus further confirming effect of these mutations on the fiber organization. Additionally, a substantial increase of desmin content in patients' muscle as well as its presence in large aggregate-like structures implies a disturbance in desmin folding and/or filament formation. Notably, the profound presence of desmin in the soluble fraction of patients' muscle, especially in this one with a point mutation, seems to confirm this suggestion. We propose that the major phenotypic differences reflect the different effects of the two mutations on desmin structure, whereas the variability of phenotypes among the patients with the A357_E359 deletion suggests an influence of environmental factors [28].

The desmin rod domain (residues 109–412) is highly conserved among intermediate filament proteins and is crucial for dimer formation, an essential early step in filament assembly. It features a seven-residue (heptad) repeat pattern *abcdefg*, where positions *a* and *d* are usually occupied by hydrophobic amino acids. Positions *b*, *c* and *f* are typically polar, whereas *e* and *g* are occupied by charged residues (in the helix 2B domain: M_a_R_b_E_c_L_d_E_e_D_f_R_g_). This alignment allows two helices to form a stable homopolymeric coiled-coil dimer, the elementary unit of the desmin filament. Segments 1A and 2B contain regions highly conserved among other intermediate filament proteins.

The heptad repeat pattern has three interruptions - linkers between the four coils: 1A, 1B, 2A, and 2B [29]. In addition, the coiled-coil segment 2B of all intermediate filament proteins contains a conserved discontinuity of the heptad repeat called a “stutter”, which is an insert of four residues (positions 356–359). This causes a local distortion of the coiled-coil geometry, however, tolerated in the coiled-coil structure. This suggests that the stutter may not only be a region in which the alpha-helices run in parallel, but that it could even represent a complete, albeit local, loss of the alpha-helical structure [30]. *In vitro* insertion manipulation of the stutter sequence (e.g. insertion of three amino acids in this “stutter” motif of vimentin) yielded a protein that assembled into unit-length filaments, but did not elongate any further [31].

Desmin filament assembly is a complex process that proceeds stepwise and begins with a gradual association of the dimers which interact laterally in an antiparallel half-staggered mode to create unit-length homodimers. Further, dimer-dimer interactions are extremely specific, and the assembly of the mature filament is therefore highly dependent on the proper folding of the elementary subunits. Next, antiparallel staggered tetramers are formed, leading to laterally associating unit length filaments that ultimately interact end to end to form the final desmin filament. The whole process of filament assembly depends on the central rod domain, whereas the tail domain is important for interactions between tetramers and elongation of higher-order filament structures [32]–[35].

The disease-associated mutations in *DES* described so far are mostly found in exon 6 (encoding amino acids 342–415, which cover most of coil 2B (spanning amino acid positions 296–412) [11]. It should be emphasized that in close vicinity of Q348P and A357_E359del numerous MFM-causing mutations are located (e.g., N342D, L345P, M349-R355del, G349ins, R350P, R350W, R355P, A357P, A360P, N366del, and L370P [11], [18], [30], [32], [36]–[40]). Most of these mutations, and seemingly the ones decribed herein impair the desmin dimer assembly, which results in disruption of the filament network and, consequently, leads to the accumulation of chimeric intracellular aggregates composed of misfolded desmin, to which other cytoskeletal proteins bind [41], [42]. Also, in other IFs proteins mutations in the coiled-coil region can lead to ectopic protein aggregation [43], [44]. Mutations in other regions, especially those in the tail domain responsible for strain stiffening [35], can also be pathogenic, e.g., I451M does not impair filament assembly but the interaction of mutated desmin with other intermediate filaments [45]. The desmin-related phenotypes are in most cases inherited in a dominant pattern.

Proline is rarely found in the heptads of coiled-coil structures, including the rod domain of IF proteins. It is a known α-helix disruptor as it imposes severe steric and hydrogen bonding constraints on the secondary structure. As a secondary rather than primery amine it is able to cause kinks in protein structure. While absent in the coil segments of desmin, proline is found in the linkers between the domains: 1A and 1B (at position 148), 1B and 2A (at position 268) and after coil 2B (at position 419). The Q348P mutation occurs at the *g* position within a heptad repeat (*abcdefg*) of the rod domain. The introduction of a helix disruptor (Pro) instead of a Gln suggest that this mutation, in a manner comparable to the L345P and R355P mutations, interferes with the desmin dimerization and filament formation. The substantial amount of misfolded desmin, especially of the patient with Q348P mutation seems to support this suggestion. Notably, proline residue is located in most cases in 2B rod domain causing myopathies.

Both modifications of desmin structure studied here, the point mutation and deletion, introduce a higher flexibility to the structure compared to the wild-type protein. Especially, the Q348P mutation increases the range of the helix bending angle from ca. 160°–180° to ca. 145°–180° (Figure 10). The presence of a local maximum for this angle at 158° for the mutated desmin indicates a possibility to form bent filaments which may lead to aggregated structures . On the other hand, the A357_E359del mutation does not introduce proline, in which it resembles other *DES* mutations causing less severe structural effects and characterized by incomplete penetrance (e.g., R350W) [38]. Indeed, images of muscle fibers with the A357_E359del mutation showed a less pronounced disruption of desmin, as compared to the Q348P muscle, where large desmin-containing aggregates were visible. Also the overall myofibrillar organization was more heavily disrupted in the Q348P muscle.

The same average number of salt bridges in all performed MD simulations indicates that the modified desmin dimers may demonstrate their abnormal behavior by increased flexibility of the backbone.

The flexibility of the A357_E359del protein is similar to that of the WT, however, the mismatch in the helix-helix interactions introduced by the deletion of three amino acid residues bringing same-sign charged residues to close proximity, could result in partial unwinding, but not bending, of the coiled-coil conformation, and thereby impair the formation of regular intermediate filaments. It is also plausible that the altering of the heptad periodicity within a critical 2B coiled-coil segment, which adds a second stutter immediately downstream of the native stutter could impair desmin folding, thereby affecting the IF assembly. The changes either in the structure of unit-dimers and polymeric IF or protein-protein interactions and cellular scaffold organization could result in MFM pathology.

All in all, we presented two heterozygous DES variants in three families with desminopathy. Based on extensive in silico and ex vivo analyses, Q348P and A357_E359del variants may be assumed pathogenic mutations. It could be speculated that abnormal structure of mutated desmin results in aberrant folding and aggregation, triggering myofibrils disruption. This study broadens the variant spectrum of desminopathy and provides evidence that mutations located in the coiled-coil segment 2B of the DES protein disturb the structure of unit-dimers and polymeric IF or protein-protein interactions between DES and its protein partners, resulting in MFM pathology.

## Supporting Information

S1 Figure MRI of the hamstring muscles of patient IV:44 from the KP family, with characteristic patterns of predominant involvement of the semitendinosus (1a) and iliopsoas (1b) muscles with relative sparing of the semimembranosus (C) and biceps femoris(D).
(DOCX)

S1 Table Clinical details of the patients.
(DOCX)

S2 Table Extended haplotype analysis of families with the *DES* mutations. Microsatellite markers in patients with the *DES* mutations are shown. Numbers indicate length (bp) of each PCR fragment. Shared alleles are presented in bold. Red background indicates common alleles for all the patients bearing del. Green background shows alleles found in family members without mutation. Orange background indicates any discrepancies, most likely due to crossing-over event (vertical double lines show the putative position of c/o). White background for the control - patient with Q348 point mutation.
(DOCX)
