# Supplementary figures and images for: Two Desmin Gene Mutations Associated with Myofibrillar Myopathies in Polish Families
Source: PLoS One. 2014 Dec 26;9(12):e115470. doi: 10.1371/journal.pone.0115470 (PMC4277352; doi:10.1371/journal.pone.0115470)

Figure S1a


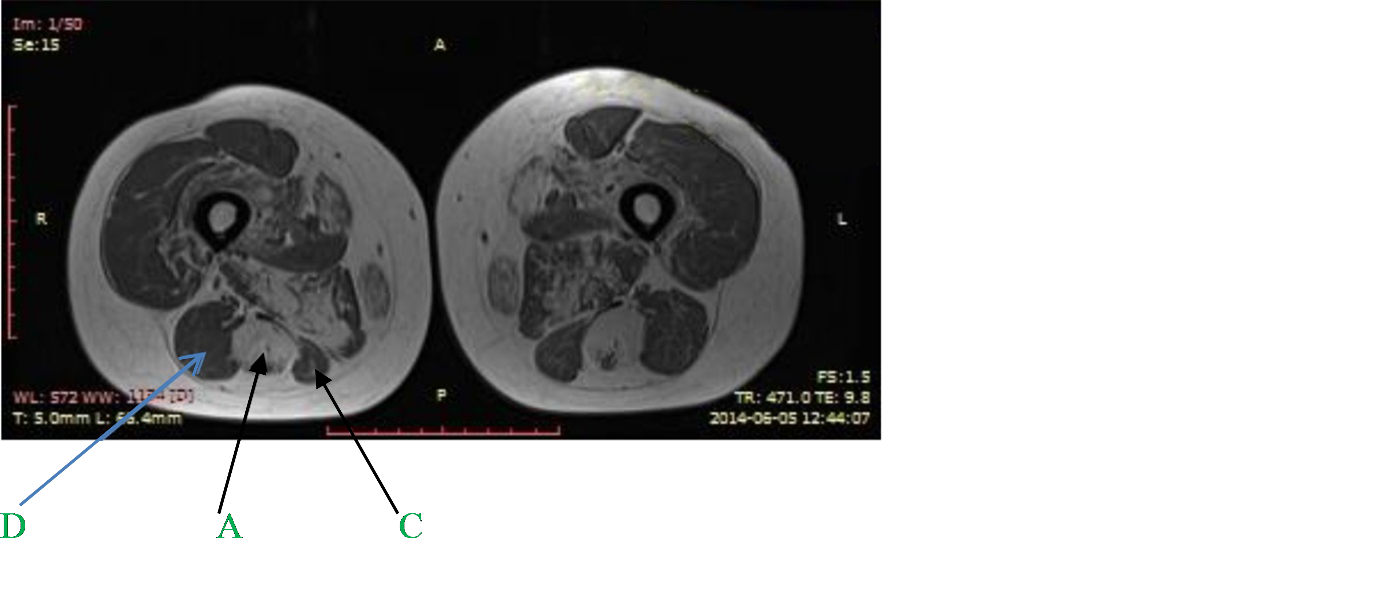


FIgure S1b


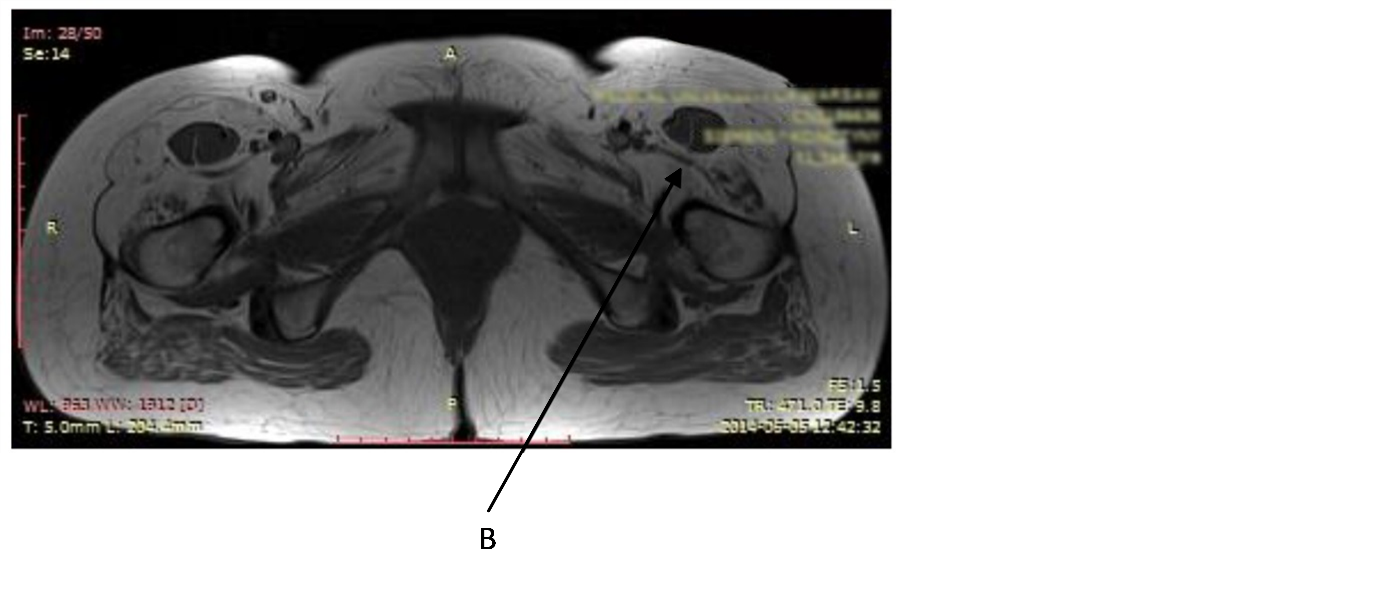

Supplement: S1 Figure — MRI of the hamstring muscles of patient IV:44 from the KP family, with characteristic patterns of predominant involvement of the semitendinosus (1a) and iliopsoas (1b) muscles with relative sparing of the semimembranosus (C) and biceps femoris(D). (DOCX) [file pone.0115470.s001.docx]
