# Supplementary material for: Two Desmin Gene Mutations Associated with Myofibrillar Myopathies in Polish Families
Source: PLoS One. 2014 Dec 26;9(12):e115470. doi: 10.1371/journal.pone.0115470 (PMC4277352; doi:10.1371/journal.pone.0115470)
Supplement: S1 Table — Clinical details of the patients. (DOCX) [file pone.0115470.s002.docx]

Table S1. Clinical details of the patients

| **Family KP** | | | | | | | | | | | |
| --- | --- | --- | --- | --- | --- | --- | --- | --- | --- | --- | --- |
| **No** | **Patient** | **Sex** | **Age** | **Age at onset** | **First symptoms** | **Neurological examination** | **Ability to walk** | **CK** | **EMG** | **Cardiac involvement/pulmonary involvement** | **Biopsy No** |
| 1 | III:11 | F | 71 | 45 | Gait disorders | Weakness Proximal>distal Upper>lower | Lost at 63 | Normal | Myopathic | None | 4/82 biceps |
| 2 | IV:40 | F | 42 | 39 | - | Mild weakness Proximal>distal Upper>lower | Normal | Normal | Myopathic | None | 118/00 biceps |
| 3 | IV:25 | F | 44 | 39 | Gait disorders | Moderate weakness Proximal<distal Upper<lower | Not on heels | Slightly elevated | Myopathic /Neurogenic NCV normal | None |  |
| 4 | IV:16 | M | 46 | 41 | Gait disorders | Moderate weakness Proximal=distal Upper<lower | Not on heels | Elevated | Myopathic | Supraventricular arrhythmia | 118/01 biceps |
| 5 | IV:44 | F | 49 | 40 | Gait disorders | Moderate weakness Proximal>>distal Upper<lower | Not on heels | Elevated | Myopathic | None |  |
| 6 | IV:6 | M | 49 | 37 | Gait disorders | Moderate weakness Proximal>>distal Upper<lower | Not on heels | Elevated | Myopathic | Not performed |  |
| 7 | IV:20 | M | 52 | 41 | Gait disorders | Mild weakness Proximal>distal Upper>lower | Normal | Slightly elevated | Myopathic | None |  |

| **Family DP** | | | | | | | | | | | |
| --- | --- | --- | --- | --- | --- | --- | --- | --- | --- | --- | --- |
| 7 | III:3 | M | 24 | 21 | Fasciculation, muscle spasm | Moderate to severe weakness Proximal<distal Upper<lower | Severe gait difficulties at the age of 28 | Slightly elevated | Myopathic | None | 44/10 vastus lateralis |
| 8 | II:2 | F | 47 | 41 | Pain in legs, gait disorders | Moderate weakness Proximal<distal Upper<lower | Not on heels | Elevated | Myopathic, decreased CMAP in both peroneal nerves | None |  |
| **Family ZP** | | | | | | | | | | | |
| 13 | IV:2 | F | 46 | 41 | Progressive gait disorders and occasional fails | Mild weakness Proximal>distal Upper<lower L>P | Not on heels | Slightly elevated | Myopathic | None | 47/10 vastus lateralis |
| 14 | IV:16 | F | 46 | 41 | Progressive gait disorders | Mild weakness Proximal>distal Upper<lower L>P | Not on heels | Slightly elevated | Myopathic | None |  |
