# Supplementary material for: Two Desmin Gene Mutations Associated with Myofibrillar Myopathies in Polish Families
Source: PLoS One. 2014 Dec 26;9(12):e115470. doi: 10.1371/journal.pone.0115470 (PMC4277352; doi:10.1371/journal.pone.0115470)
Supplement: S2 Table — Extended haplotype analysis of families with the DES mutations. Microsatellite markers in patients with the DES mutations are shown. Numbers indicate length (bp) of each PCR fragment. Shared alleles are presented in bold. Red background indicates common alleles for all the patients bearing del. Green background shows alleles found in family members without mutation. Orange background indicates any discrepancies, most likely due to crossing-over event (vertical double lines show the putative position of c/o). White background for the control - patient with Q348 point mutation. (DOCX) [file pone.0115470.s003.docx]

Table S2. Extended haplotype analysis of families with the *DES* mutations.

Microsatellite markers in patients with the DES mutations are shown. Numbers indicate length (bp) of each PCR fragment. Shared alleles are presented in bold. Red background indicates common alleles for all the patients bearing del. Green background shows alleles found in family members without mutation. Orange background indicates any discrepancies, most likely due to crossing-over event (vertical double lines show the putative position of c/o). White background for the control - patient with Q348 point mutation.

|  | **D2S2242** | **D2S2208** | **D2S2382** | **D2S2248** | **D2S1338** | ***D2S163*** | ***DES*** | **D2S126** | **D2S133** | **D2S2354** | **D2S2297** | **D2S2317** |
| --- | --- | --- | --- | --- | --- | --- | --- | --- | --- | --- | --- | --- |
| ZP IV:2 Patient | 195 **210** | **136** 138 | **308** | **182** | **238** 250 | 220 **222** | **del** | **125** 139 | **240** | 262 276 | **242** 246 | 318 **319** |
| ZP IV:4 Patient | 208 **210** | 127 **136** | **308** | **182** 193 | **238** 250 | 220 **222** | **del** | **125** 139 | **240** | 262 276 | **242** 256 | **319** 328 |
| KP III:11 Patient | 206 **208** | 129 **135** | **308** 332 | **182** 195 | **238** 266 | 220 **222** | **del** | **125** 141 | **240** 242 | 262 **266** | **256** | 324 **328** |
| KP IV:40 Patient | 202 **208** | **135** 139 | **308** | **182** 190 | **238** | **222** 228 | **del** | **125** 135 | **240** | **266** 276 | 242 **256** | 319 **328** |
| KP IV:44 Patient | 204 **208** | 127 **135** | **308** 322 | **182** 193 | 234 **238** | **222** 226 | **del** | **125** | **240** 242 | 262 **266** | 235 **256** | 322 324 |
| KP IV:25 Patient | 198 206 | 129 145 | **308** | **182** | **238** | **222** 226 | **del** | **125** 135 | 232 **240** | 262 **266** | 240 **256** | 318 330 |
| KP V:43 Healhy | 202 208 | 127 135 | 308 | 190 197 | 238 270 | 218 228 | WT | 135 | 240 242 | 266 276 | 242 | 302 319 |
| KP V:44 Healthy | 200 202 | 127 139 | 308 | 190 197 | 238 270 | 218 228 | WT | 135 | 240 242 | 266 276 | 242 252 | 319 328 |
| KP III:1 Patient | 200 **202** | **141** 156 | **308** | **182** | **238** 266 | **222** 224 | **del** | **125** 146 | **240** | 262 **266** | 250 **256** | 319 **328** |
| KP V:7 Healthy (pre-onset) | **202** 206 | 129 **141** | **308** 318 | **182** | **238** 270 | **222** | **del** | **125** 135 | **240** 244 | **266** 270 | 250 254 | 319 **328** |
| KP V:8 Healthy (pre-onset) | **202** 210 | **141** 147 | **308** 338 | **182** | **238** 242 | **222** 226 | **del** | **125** | 238 **240** | **266** 270 | 254 **256** | 326 **328** |
| KP V:9 Healthy | 200 210 | 147 156 | 308 338 | 182 | 241 266 | 224 226 | WT | 125 146 | 238 240 | 262 270 | 250 | 318 319 |
| KP IV:20 Patient | 202 208 | 131 135 | 320 330 | 182 192 | 250 274 | **222** 228 | **del** | **125** 141 | 236 **240** | **266** 272 | 242 **256** | 302 **328** |
| DP III:3 Patient | 195 204 | 127 137 | 308 | 182 | 238 266 | 224 230 | Q348P | 141 148 | 240 | 262 264 | 250 254 | 306 328 |
